# Telomere Length and Oxidative Stress and Its Relation with Metabolic Syndrome Components in the Aging

**DOI:** 10.3390/biology10040253

**Published:** 2021-03-24

**Authors:** Graciela Gavia-García, Juana Rosado-Pérez, Taide Laurita Arista-Ugalde, Itzen Aguiñiga-Sánchez, Edelmiro Santiago-Osorio, Víctor Manuel Mendoza-Núñez

**Affiliations:** 1 Research Unit on Gerontology, FES Zaragoza, National Autonomous University of Mexico, Mexico City 09230, Mexico; ggg1501@hotmail.com (G.G.-G.); juanarosadoperez@comunidad.unam.mx (J.R.-P.); tdlarista@gmail.com (T.L.A.-U.); 2 Hematopoiesis and Leukemia Laboratory, Research Unit on Cell Differentiation and Cancer, FES Zaragoza, National Autonomous University of Mexico, Mexico City 09230, Mexico; itzen.aguiniga@zaragoza.unam.mx (I.A.-S.); edelmiro@unam.mx (E.S.-O.)

**Keywords:** metabolic syndrome, telomere, telomerase, oxidative stress, aging, lifestyles

## Abstract

**Simple Summary:**

A link between telomere length and some age-related diseases has been identified, including metabolic syndrome. So far, there is no mechanism to explain the origin or cause of telomere shortening in this syndrome; however, oxidative stress is a constant factor. Therefore, we reviewed scientific evidence that supported the association between oxidative stress and telomere length dynamics, also examining how each of the metabolic syndrome components individually affects the length. In this regard, there is strong scientific evidence that an increase in the number of metabolic syndrome components is associated with a shorter telomere length, oxidative damage at the lipid and DNA level, and inflammation, as well as its other components, such as obesity, hyperglycemia, and hypertension, while for dyslipidemia, there is a little more discrepancy. The difficulty for the correct treatment of metabolic syndrome lies in its multifactorial nature. Hence, there is a need to carry out more studies on healthy lifestyles during aging to prevent and reduce oxidative damage and telomere wear during aging, and consequently the progression of chronic degenerative diseases, thus improving the living conditions of older people.

**Abstract:**

A great amount of scientific evidence supports that Oxidative Stress (OxS) can contribute to telomeric attrition and also plays an important role in the development of certain age-related diseases, among them the metabolic syndrome (MetS), which is characterised by clinical and biochemical alterations such as obesity, dyslipidaemia, arterial hypertension, hyperglycaemia, and insulin resistance, all of which are considered as risk factors for type 2 diabetes mellitus (T2DM) and cardiovascular diseases, which are associated in turn with an increase of OxS. In this sense, we review scientific evidence that supports the association between OxS with telomere length (TL) dynamics and the relationship with MetS components in aging. It was analysed whether each MetS component affects the telomere length separately or if they all affect it together. Likewise, this review provides a summary of the structure and function of telomeres and telomerase, the mechanisms of telomeric DNA repair, how telomere length may influence the fate of cells or be linked to inflammation and the development of age-related diseases, and finally, how the lifestyles can affect telomere length.

## 1. Introduction

### 1.1. Structure and Function of Telomeres

Telomeres are DNA-protein complexes that are localised at the edge of chromosomes, with more than 2000 repetitions of the “TTAGGG” sequence of non-coding double-strand DNA and ending with a guanine rich single-stranded DNA [1,2]. Telomeres are necessary for the stability and protection of genomic DNA and prevention of chromosomal fusion [3]. In addition, they are involved in signaling pathways that regulate cell proliferation, thus establishing the lifespan of a cell [4,5]. Hence, telomeres are considered biological clocks that determine the number of divisions that a cell undergoes [6,7]. Telomeres are constituted by protein complexes such as the telosome or shelterin complex, CTC1-STN1-TEN1 (CST) complex, and associated proteins [8,9], whose general function is to guide the cell fate through dynamic structural and organisational transitions [10].

Shelterin is a protein complex consisting of six subunits: telomeric repeat-binding factor 1 (TRF1), telomeric repeat-binding factor 2 (TRF2), protection of telomeres protein 1 (POT1), repressor activator protein 1 (RAP1), TRF1-interacting nuclear factor 2 (TIN2), and adrenocortical dysplasia protein homolog (TPP1) [10]. Meanwhile, the CST complex is comprised of three proteins: conserved telomere protection component 1 (CTC1), suppressor of cdc thirteeN 1 (STN1), and telomeric pathway with STN1 (TEN1) [11]. Shelterin complex binds to the telomeres through TRF1 and TRF2. TRF1 is involved in the negative regulation of the telomeric length (TL), by inhibiting the telomerase activity [12,13,14]. On the other hand, TRF2 plays an important role in the cell cycle progression and the protection against the chromosome end-to-end fusion. Both proteins present three helixes that bind exclusively to the telomeric DNA through a Myb-type domain. The third helix of TRF1 recognises the TT sequence and binds to the DNA as a homodimer, preferably to double-stranded DNA; then, it binds with TIN2, which confers the capacity to interact with TPP1 and POT1. On the other hand, the TRF2 homodimer binds to double-stranded DNA and then, similar to TRF1, it binds to TIN2 [14,15,16].

POT1 interacts directly with the single-stranded DNA and can tangle with TPP1. This protein plays different roles including avoiding segregation defects, chromosomic instability, loss of telomeric sequences, chromosome end-to-end fusion, and unwanted repairing activities [17,18,19]. Regarding the RAP1 subunit, it is known that this protein is characterised for its dual function, as it participates in the control of gene expression, as well as in chromosome maintenance [20]. In addition, it can interconnect with TRF2 [21].

TIN2 is another important protein of the shelterin complex that binds simultaneously to POT1/TPP1 and TRF1/TRF2, and it avoids the wrong identification of damage in the telomeric DNA [21,22] and, similar to TRF1, it can act as a negative regulator of the TL [4,14]. The TPP1 protein can assemble with the telomeres through POT1 and TIN2 [23,24], and it also takes part as a recruitment factor for the telomerase enzyme through a region known as the strictly conserved surface of TPP1 OB (TEL patch) [25] as well as in the stimulation of the enzyme progression [26].

**Figure 1:**
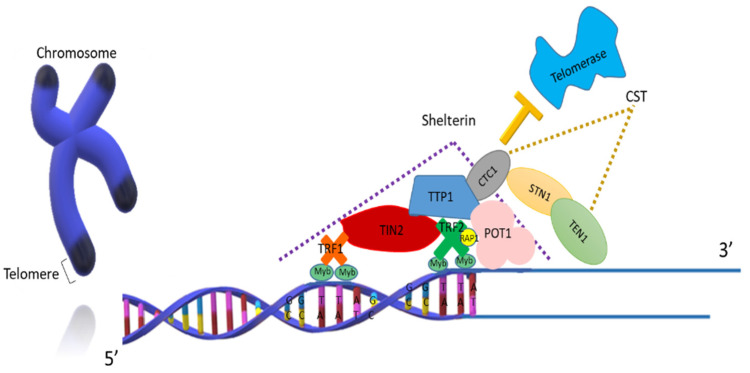
Structure of the shelterin and CTC1-STN1-TEN1 (CST) complexes. The shelterin complex is made up of the proteins telomeric repeat-binding factor 1 (TRF1), telomeric repeat-binding factor 2 (TRF2), TRF1-interacting nuclear factor 2 (TIN2), adrenocortical dysplasia protein homolog (TPP1), protection of telomeres protein 1 (POT1), and repressor activator protein 1 (RAP1). TRF1 and TRF2 subunits bind to the double-stranded telomeric DNA using Myb-type domains and the POT-1 subunit binds to the single-stranded. On the other hand, the CST complex made up of the proteins CTC1, STN1, and TEN1 inhibits the activity of the telomerase enzyme.

The synthesis of the complementary chain through the DNA polymerase α is necessary to achieve telomere homeostasis [27]. However, this enzyme is not able to perform this function without the participation of the single-strand DNA binding complex CST. This protein complex promotes the duplex replication of the telomeres; that is why its elimination leads to dysfunction, instability, and telomeric frailty [28,29]. The interaction of the proteins CTC1 and STN1 limits the action of the telomerase to prevent the overextension of the guanine rich single-stranded DNA overhang; hence, it is considered as a regulatory step that limits telomere elongation [30,31]. On the other hand, TEN1 is a crucial protein for the synthesis of the complementary chain, since its absence triggers alterations in the replication restart; likewise, it participates in the telomere maintenance and protection and is a negative regulator of the telomerase enzyme (Figure 1) [31,32,33]. Finally, without the protective activity of each of these complexes, the DNA damage repair mechanisms could wrongly process the edge of the chromosomes [34].

### 1.2. Telomerase Structure and Function

Every time a cell divides, about 25–200 bp of the telomeric sequence is lost, which is a phenomenon that is also known as “the end-replication problem” where DNA polymerase cannot complete the terminal space of the double helix [35,36]. Hence, with the purpose of properly making the replication happen, the telomerase enzyme is required, which is a ribonucleoprotein that helps in the maintenance of the telomere length, in the 3’-OH chromosome end, through the transport and addition of tandem repeat RNA templates [37]. It has been established that when the telomeres are long enough, the presence of telomerase is not required, being necessary only when the TL falls within a critical range [38]. The human telomerase is constituted of two subunits that work simultaneously: the human telomerase RNA (hTERC, hTR, or TER) and the human telomerase catalytic subunit (hTERT) [39]. hTERC is an RNA molecule that is necessary for the stability and processing of the 3′ of the DNA; it contains a short complementary template of the telomeric DNA sequence [40]. hTERT has a reverse transcriptase activity and is responsible for adding the new segment without altering the phenotypic and morphogenic characteristics of the cell [41]. Subsequently, telomerase uncouples, and the DNA polymerase begins the synthesis of the complementary fragment of the 5′ end, avoiding then the telomeric shortening that comes with every cell division [42].

It is to notice that other accessory proteins are required for the telomerase to be fully functional, including dyskerin pseudouridine synthase 1 (DKC1), which is involved in telomere maintenance, ribosomal RNA biosynthesis, and pseudouridylation [43], and the telomerase Cajal body protein 1 (TCAB1), which is capable of associating with the telomerase and directly moving it towards the telomere. It is known that a TCAB1 depletion impedes hTERC from binding to Cajal bodies (nuclear regions that participate in the organisation of the genome, genic expression and RNA splicing fidelity) [44,45,46]. Some proteins are located in the Cajal bodies and can interact with the hTERC subunit [47] denominated: H/ACA ribonucleoprotein complex subunit 1 (GAR1), H/ACA ribonucleoprotein complex subunit 2 (NHP2), and H/ACA ribonucleoprotein subunit 3 (NOP10), considered key biomolecules of the small nucleolar RNAs (snoRNAs); thus, these proteins participate in post-transcriptional mechanisms such as methylation and ribosomic RNA modifications [48]. It has been observed that patients that present mutations in NHP2 or NOP10 present low levels of hTERC subunit in peripheral blood, which leads to alterations in the telomere [49].

**Figure 2:**
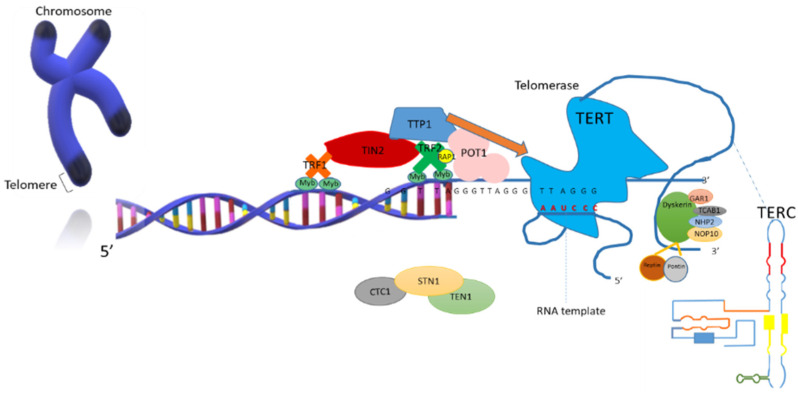
Scheme of the telomerase complex, consisting of two main subunits: telomerase RNA (TERC) that contains the RNA template region, and telomerase catalytic subunit (TERT), which is responsible for adding the new segment. Adrenocortical dysplasia protein homolog (TPP1) acts as a telomerase recruiter. In addition, the accessory proteins for the enzyme to be fully functional are shown: Dyskerin, H/ACA ribonucleoprotein complex subunit 1 (GAR1), telomerase Cajal body protein 1 (TCAB1), H/ACA ribonucleoprotein complex subunit 2 (NHP2), and and H/ACA ribonucleoprotein subunit 3 (NOP10). The pontin and reptin proteins are in charge of its remodeling and assembly.

Other proteins that play an essential role in the assembly or remodeling of the telomerase are ATPases pontin and reptin (Figure 2) [50,51]. Both proteins participate in telomerase biogenesis, particularly in the S phase of the cell cycle. The reptin/pontin/TERT complex is considered an immature complex, also known as pre-telomerase with low catalytic activity, which can subsequently rearrange to form the mature TERC/TERT/DKC1 complex with a high catalytic activity [52].

The activity of the telomerase enzyme is limited in most somatic cells, causing the telomeres to shorten, which finally leads to replicative senescence (an irreversible arrest of the cell cycle). However, the activity of this enzyme can be reactivated in between 80 and 90% of the distinct types of cancer. There is no evidence of another broadly expressed gene among all known cancers [38,53,54]. In addition, 5–10% of the tumors use a molecular mechanism denominated the alternative lengthening of telomeres (ALT) that provides the capacity of maintaining the telomere length even without telomerase [54,55]. The carcinogenic transformation also seems to depend on certain types of responses such as oncogenic activation or tumor suppressors inactivation such as p53 [41]. Similarly, the elongation of the TL is related to the immortal cell phenotype, which is considered as a hallmark of neoplasic cells [56]. Thus, the activity of this enzyme can be used as a key diagnostic tool for cancer, while its inhibitors could be used as antitumoral agents [6,57].

On the other hand, researchers have determined an association between the levels and/or the activity of the telomerase and metabolic disorders such as type 2 diabetes mellitus (T2DM), hypertension, dyslipidemia, and psychological stress [58,59]. For example, in animal models, it has been identified that a lower telomerase activity caused by a TERC subunit deficiency affects the capacity of replication of β-pancreatic cells, causing alterations in insulin secretion and glucose intolerance [58]. Conversely, it has been suggested that non-controlled hypertension is associated with high telomerase activity in leukocytes [60]. Based on the above, it can be proposed that metabolic disorders are key factors that can trigger the dynamics of telomerase activity behavior. In addition, it is important to highlight that many metabolic disorders are associated with changes in TL in aging [61].

## 2. Telomeric Length and Cell Fate in Aging

Normally, when telomeres reach a critically short length in one or more chromosomes, they no longer have the capacity to safeguard the genome integrity [62]. For example, it is estimated that the minimal number of repetitions for the correct maintenance of the telomere must ≥500 bp [63], and at least 12.8 TTAGGG repetitions prevent chromosomes from end-to-end fusion [64]. This leads the cell to limit the proliferation through processes such as senescence, which is a mechanism that permanently blocks cell division, and apoptosis (programmed cell death) [57,65,66].

During aging, there is a major drawback when TL is shortened, as organ regeneration capacity is limited with possible modifications in gene expression even before the onset of senescence or apoptosis [67,68]. Senescent cells are generally eliminated from the tissue by the immune system. However, the impaired immune system of aged individuals fails to clear all the senescent cells; thus, the ones that remain in the tissue are non-functional cells that can jeopardise the tissue architecture and function [7]. In other words, senescent cells are accumulated through aging and in some cases promote the appearance of cancer in the tissue [68,69,70,71,72].

The above can be attributed to the fact that senescent cells are metabolically active and can influence the surrounding cells through the secretion of growth factors, cytokines, chemokines, and matrix-remodeling enzymes, also known as Senescence-Associated Secretory Phenotype (SASP), which can be associated with the structural and functional decay of the tissue, also triggering tumorigenesis [71].

There are new encouraging strategies to attenuate health decline at older ages or to delay aging. One of them is through the elimination of senescent cells by compounds called senolytics or by senomorphic compounds that inhibit or attenuate the SASP [73,74]. The research on such molecules has been growing in recent years, and therefore, the association between senolytics and senomorphics with other age-related diseases has come to light. Regarding the latter, it has been observed that the acute administration of lithium carbonate is able to reduce the SASP of human astrocyte-derived induced pluripotent stem cells (iPSCs) [75]. In addition to the above, the administration of this compound is also involved in telomere length dynamics, since chronic treatment in a triple transgenic experimental model of Alzheimer’s disease increases TL in the hippocampus and parietal cortex in a tissue-specific manner [76]. Furthermore, its long-term use is associated with longer TL in peripheral blood leukocytes from bipolar patients [77]. Therefore, pharmacological targeting of these compounds could transform geriatric medicine by preventing or delaying telomeric attrition and/or the onset of metabolic diseases such as metabolic syndrome (MetS), where senescence could be contributing by increasing systemic inflammation and insulin resistance in T2DM [74,78,79,80,81]. Although the effect of senolytics has been proven, we should not lose sight of the fact that senescence also participates in beneficial processes for health, such as tissue repair and protection against the development of carcinogenic processes, among others [82].

Telomeres do not trigger apoptosis by themselves, but the absence of the protein TRF2 in the shelterin complex does trigger this process because the DNA without a telomere is identified as damaged, which is a process regulated by proteins such as p53 and the kinase Ataxia Telangiectasia Mutated (ATM) [83,84].

Telomeric shortening is not exclusive of somatic cells; it can also occur in gametic cells. For example, in human oocytes, telomere shortening leads to apoptosis in embryonic cells [85], being more frequent in aged women, with the possibility of miscarriage, birth defects, and infertility [86]. On the other hand, TL shortening in spermatocytes is evident in men with a low concentration of spermatocytes (oligozoospermia) that is directly associated with the age of the parents at the moment of conception [87], which predispose to apoptosis in spermatic cells [88] and male infertility [89].

## 3. Reactive Oxygen Species and Antioxidants

The mitochondrion plays a crucial role for cell survival, as it is involved in ATP production by electron transport enzyme complexes in the respiratory chain, in which reactive oxygen species (ROS), such as superoxide radical (O_2_^●−^) are invariably produced by electron transfer to molecular oxygen. This happens particularly in complexes I (Nicotine adenine dinucleotide reduced (NADH) ubiquinone oxidoreductase) and III (ubiquinol–cytochrome c oxidoreductase) of the mitochondrial inner membrane [90].

Under physiological conditions, ROS act as second messengers that participate in several signaling pathways to maintain the homeostasis, growth, and normal development of aerobic organisms [91,92]. However, Oxidative Stress (OxS) occurs when there is an imbalance of oxidants (such as ROS) and antioxidant mechanisms, where the presence of the first is in a higher proportion [93,94]. As mentioned earlier, ROS such as the O_2_^●−^ are generated by the electron transport chain and, in the presence of transition metals such as Fe^3+^, transforms into hydrogen peroxide (H_2_O_2_), which together with O_2_^●−^ becomes hydroxyl radical (^●^OH) through Fenton and Haber–Weiss reactions [95]. Hydroxyl radical and oxygen singlet (^1^O_2_) can generate DNA adducts, such as thymine glycol or 8-hydroxy-2′-deoxyguanosine (8-OHdG). The last one results from the binding of the ^●^OH to the eighth carbon of the guanine [96]. 8-OHdG transforms into its oxidised form 8-oxo-7,8-dihydro-2′-deoxyguanosine (8-oxodG) through a keto-enol tautomerism reaction, which is considered the most abundant oxidative injury, with about 100,000 8-oxodG generated every day in a single cell [97,98].

Despite all the damage that elevated ROS levels may induce, the organisms are endowed with antioxidant defense mechanisms, which are synthesised de novo (endogenous) or acquired through the diet (exogenous). These systems are capable of counteracting or eliminating directly or indirectly the ROS and/or its derivatives, such as reactive nitrogen species (RNS) or reactive sulfur species, to maintain redox homeostasis [99,100]. For example, O_2_^●−^ can be rapidly transformed by the enzyme superoxide dismutase (SOD) into H_2_O_2_, which is converted into less toxic products by the enzyme catalase (CAT) or glutathione peroxidase (GPx) [90]. However, oxidative damage increases gradually over time because antioxidant defense mechanisms decline with age [101]. It has been proposed that OxS theory is a direct cause for aging, which is based on the molecular disorder generated by the free radical accumulation [102]; this disorder may cause oxidative damage in several biomolecules and alterations in redox homeostasis, resulting in the appearance of age-related diseases [103,104,105,106].

Likewise, OxS can influence cellular fate, since it has been proposed that ROS are triggers of senescence and apoptosis. For example, it has been observed that fibroblasts treated with low concentrations of H_2_O_2_ become senescent, while treatment with a high concentration leads to apoptosis [107]. Other in vitro studies have revealed that cells with a normal TL and telomerase expression show greater resistance against apoptosis compared to cells from older organisms [108]. Thus, higher concentrations of ROS upregulate the signaling pathways associated with cell death [109,110].

## 4. Telomere Shortening and Oxidative Stress

Due to the high content of guanines in the telomeric region, it is susceptible to oxidative damage. Thus, the adduct can be found in this region at a higher proportion, even seven times more, compared to genomic DNA [111,112]. The molecule 8-oxodG is considered as a pre-mutagenic lesion that is capable of inadequately binding to adenine, leading to transversions GC-TA, which subsequently generates several alterations such as single-strand brakes, inadequate replication of the telomeric DNA, accelerated telomere shortening [113], and if the DNA damage repair mechanisms fail, it can lead to cellular physiology modifications, senescence, or apoptosis [91].

It has been postulated that even without oxidative damage, the telomeres would shorten with every mitotic cycle, and the aging process would happen inevitably. However, during the aging process, OxS is a major factor that significantly contributes to the telomere shortening rate [114,115].

In addition, it has been proposed that the oxidative damage induced by ROS induces a tissue-specific reduction of the TL, which is derived from the antioxidant capacity of each tissue [116,117]. The damage generated by OxS can interfere with the assembly of the telomere maintenance proteins such as TRF1, TRF2, and POT1 with the DNA; even a single 8-oxodG lesion can induce a 50% reduction in the levels of such proteins, leading to telomeric instability [118]. Meanwhile, it has been observed that senescent cells’ 8-oxodG levels are 35% higher compared to control cells by inducing cell growth arrest [119].

Another event linking OxS and rapid telomere attrition is given by the loss of the enzyme peroxiredoxin-1 (PRDX1), where the DNA damage sensor-dependent repair involved in the base excision repair (BER) mechanism, known as poly(ADP-ribose)-polymerase-1 (PARP1), is inefficient [120]. This condition causes the accumulation of telomeric single-strand DNA breaks (SSBs), which in turn become potentially lethal telomeric double-strand breaks (DSBs) [121]. DSBs are repaired by the high-fidelity template-dependent homologous recombination (HR) repair pathway or error-prone non-homologous end joining (NHEJ) [122]. For example, alterations in HR repair generated by the inhibition of PARP1 activity cause SSBs to persist, leading to genomic catastrophe and telomeric shortening, and ultimately apoptosis [120,123].

## 5. Telomere Length and Age-Related Diseases and Oxidative Stress

Aging is defined as a gradual and adaptive process that is characterised by a decline in the biological response to maintain or recover homeostasis against the challenges that a person faces during the lifespan in a given environment [124]. It is estimated that in 2050, the percentage of people over 60 years may reach 22%. This means that in the world, there will be around 2000 million people belonging to this age group [125]. That is why it is necessary to count on opportune interventions that allow elderly people to contribute to social development and prevent them from turning into a crisis factor for healthcare structure [126].

**Figure 3:**
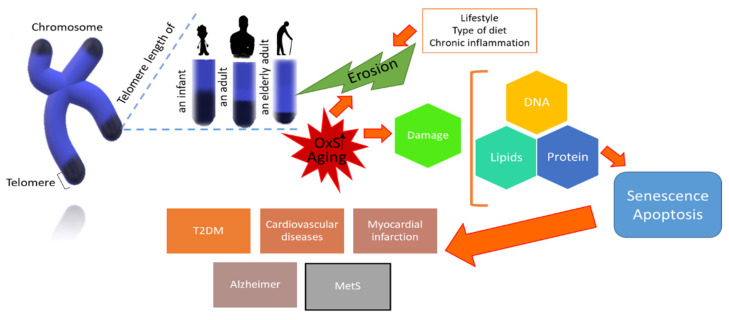
Telomere attrition in aging. OxS and other factors such as lifestyle can cause damage to biomolecules, apoptosis, or senescence, which manifests itself with the appearance of age-related diseases, among them the MetS. OxS: Oxidative Stress; MetS: Metabolic Syndrome.

It is well accepted that telomeres help stabilise the nuclear genome with high fidelity, but this function declines with age in the post-reproductive stage [1]. The loss of telomeric repetitions as well as the enzymatic activity of the telomerase are dynamic processes that regulate differentially during each stage of life [6,56]. Thus, short telomere length is a biomarker of aging that incites the cell to develop certain diseases, limiting with it the overall health status of a person [127]. Several studies support the association between a short telomere with age-related diseases and their complications, such as T2DM, cardiovascular diseases, myocardial infarction, cataractogenesis, osteoporosis, MetS, neurodegenerative diseases such as Alzheimer’s disease, and sleep disorders; as well as dementia, cognitive decline and premature mortality (Figure 3) [127,128,129,130,131,132,133,134,135]. In that same order, researchers have identified gene variations in human leukocytes, specifically a single nucleotide polymorphism (SNP) rs3772190 in the TERC locus, which is associated with a short TL and longevity [136]. It has been suggested that antioxidants could reduce the telomere shortening rate during aging, but when it comes to transformed cells, the effect may not be wanted [2,137]. To date, there has been no other inherent mechanism that explains the origin and/or causes of the telomere shortening in these pathologies. However, OxS seems to be constant [138].

There is evidence that points out that besides age, there are other factors related to telomere attrition, among them the lifestyle, diet, as well as environmental and social response to which the individual is subjected [134,139], and at a cellular level, chronic inflammation and oxidative damage [140,141], which may compromise the immune system functions [142,143]. As for OxS, it should be noted that it is associated with short telomeres in aging, which have been found in recent studies in people over 65 years old with high iron levels [144] and sarcopenia [145]. Likewise, it has been observed an increase in lipoperoxidation [146,147] is associated with a higher risk of death [148], a decay in the overall antioxidant state, and the activity of the GPx in erythrocytes from healthy people >60 years old compared to young adults. Therefore, these findings suggest that an advanced age predisposes to an increase in OxS [147].

## 6. Damage Repair of the Telomeric DNA

The habitual genomic DNA damage repair mechanisms must be modified or suppressed in the telomeric regions in order not to be recognised and processed as DSBs [55]. Telomeric DNA repair is known to be not as efficient as in other chromosomal areas, since there is evidence that shows that the genome of human cells put through OxS is repaired in the first 24 h, while the telomeric regions can remain unrepaired for up to 19 days [149]. Similarly, telomeric DNA repair is less efficient in aged cells, where it possibly has an impact on genome stability [150]. On the other hand, it has been proposed that cell senescence originates as a consequence of irreparable damage to telomeric DNA [151].

Telomeric DNA can be repaired by BER and to a lesser extent by nucleotide excision repair (NER) and mismatch repair (MMR) [152]. In general, it is speculated that BER repairs the small lesions in the DNA; on the contrary, NER eliminates the bigger ones [153]. Likewise, some products generated by oxidation, such as 8-oxodG, are identified by BER, while the 5′8-cyclopurines are recognised by NER [154]. Surprisingly, the proteins shelterin, TRF1, TRF2, and POT1 help in the repair process by increasing the speed of individual steps of the BER long-patch repair and with that protecting the telomeric DNA from degradation [155].

Oxidative damage in the DNA is repaired mainly by the enzyme 8-oxoguanosine DNA glycosylase-1 (OGG1), which is a glycosylase that has the capacity of excising directly the 8-oxodG lesion from the nuclear or mitochondrial DNA [156]. It is known that some telomeric structures such as 3′-overhang, D-Loop, and fork-opening are eliminated with lower efficiency by the OGG1 [157]. A decrease in the expression of OGG1 may lead to the accumulation of oxidant lesions associated with tumoral phenotypes [158,159]. 

Hence, a malfunction in the repair mechanisms is associated with accelerated aging and chromosome instability, which predisposes to mutagenesis, carcinogenesis, or morphological nuclear abnormalities such as nuclear buds, micronucleus, and nucleoplasmic bridges [160].

Likewise, new evidence indicates that telomeric dysfunction causes the transcription of telomeric non-coding RNAs (tncRNAs) that in turn control the DNA damage response (DDR) [161]. This transcription gives rise to a type of long noncoding RNAs (lncRNA) known as TERRA (Telomeric repeat-containing RNA) that are involved in the maintenance and processing of unprotected telomeres, chromosomal end heterochromatin formation, and telomerase activity [162,163,164,165]. Inhibition of tncRNAs by sequence-specific telomeric antisense oligonucleotides (tASOs) has been observed to prevent DDR activation [166] and cellular senescence both in vivo and in vitro, thereby improving homeostasis in fibroblasts from patients with Hutchinson–Gilford progeria syndrome (HGPS), which is characterised by premature aging and extending lifespan in an HGPS transgenic mouse model [161]. Similar to senolytics and senomorphics, inhibiting these ncRNAs with tASOs may be a promising step to prevent telomeric dysfunction, improve age-related diseases, and have a translational impact on MetS.

## 7. Relationship of Oxidative Stress with the Metabolic Syndrome and Telomeric Length

Advanced age is a risk factor for diseases that generally develop in a chronic and overlapping way—that is, they are long-lasting and associated with each other [167]. Among them is the MetS [168], whose prevalence among the youth from 20 to 29 is less than 10%; while in adults aged 60 and over, it increases by 50% [169,170].

MetS is defined as a set of biochemical and clinical alterations characterised by obesity, dyslipidemia, arterial hypertension, insulin resistance, and hyperglycemia, presenting a prothrombotic and pro-inflammatory state [171]. This syndrome is a risk factor for cognitive decline, dementia, frailty, T2DM, and cardiovascular diseases, whose pathophysiology is associated with an increase in OxS [172,173].

In the same vein, it is assumed that both aging and MetS generate OxS, but the impact of the syndrome on this imbalance is much more significant than the produced by age, since the pro-oxidant state is manifested in each of its components, predisposing to metabolic and cardiovascular complications and organ damage, especially in the elderly [174,175,176]. A positive association between MetS components and OxS levels has been reported by cumulative effects; that is, a greater presence of components leads to higher levels of OxS [177]. Currently, it is under discussion whether OxS is the cause or consequence of MetS; however, it is hypothesised that both may be true [178].

In patients with MetS, there are alterations in the antioxidant protection mechanisms and in the inflammatory process; for example, high concentrations of H_2_O_2_ decreased the gene expression of SOD and increased the levels of interleukin 1 beta (IL1β) in peripheral blood mononuclear cells (PBMCs) [179]. Similarly, an increase in transition metals such as iron or copper can generate OxS and exacerbate it, since high concentrations of these metals in serum [180,181] are associated with obesity [182], hypertriglyceridemia, low high-density lipoprotein-cholesterol (HDL-C) levels [183], hypertension [184], blood glucose, and increased predisposition to T2DM [185].

Likewise, the presence of this syndrome predisposes to an increase in free radicals (FR), which are accepted as underlying mechanisms for mitochondrial dysfunction and an accumulation of oxidation products at the level of DNA (8-oxodG), lipids (prostaglandin F2 alpha (PGF2α), malondialdehyde (MDA) and 4-hydroxynonenal (HNE) [186,187,188], carbonylated proteins (PCO) [189], low-density lipoproteins (LDL) [190], and carbohydrates (glyoxal and methylglyoxal) [174,191], which are associated with a decrease in antioxidant defense mechanisms due to low levels of vitamins C, E, and carotenoids [192,193,194], reduced glutathione (GSH) [195] and the enzymatic activity of SOD, GPx [196], and CAT [189]. This redox imbalance leads to an increased risk of metabolic complications [197,198] and acceleration of telomeric attrition in the pathogenesis of MetS [199] (Table 1). The consequences of MetS at the telomere length level can be so serious that it can even affect the offspring, since the children of mothers with MetS may have shorter telomeres [200], which predisposes to a greater risk of presenting some chronic non-communicable diseases and mortality [201].

On the other hand, MetS has been positively associated with an increase in telomerase levels and activity [189,202] and inversely with oxidised low-density lipoprotein (Ox-LDL), which leads to senescence and risk of chronic diseases such as atherosclerosis [190,203] or, in the worst scenario, to the development of malignant tumors [204], since this condition is associated with a higher risk of colorectal cancer, as well as salivary glands and mortality from breast cancer in women [205,206]. In addition, in men, there is an increased risk of pancreatic cancer and Hodgkin’s lymphoma of the thyroid [206]. Hence, the increasingly apparent link between MetS and cancer.

Similarly, there is information linking MetS components or OxS to telomere length in in vivo and in vitro models. For example, in a chimeric mouse model in which 100% of its cells were derived from embryonic cells with longer telomeres than normal (hyperlong telomeres) and lower levels of LDL and cholesterol, better tolerance to glucose and insulin were observed, and they are evidently thinner compared to mice of the same genetic origin as well as associated with a lower incidence of cancer and greater longevity [207]. In addition, in sheep and human fibroblasts under prooxidant or antioxidant culture conditions, a positive correlation was found between the levels of DNA damage by OxS and telomere shortening rates, independent of the donor [62]. Likewise, high levels of glucose together with a persistent proinflammatory state results in a higher rate of telomere erosion in aged human fibroblasts [208]. In human fibroblasts under hyperglycemia conditions, TL decreases [209], while caloric restriction in leukocyte and skin samples from rhesus monkeys apparently does not affect telomere length [210].

## 8. Components of the MetS and Its Relationship with Oxidative Stress and Telomeric Length

A great variety of studies support that each of the characteristic MetS conditions is related to OxS. For example, it is well established that fat accumulation is associated with OxS at the systemic level, which is mainly due to a decrease in antioxidant enzymes and an increase in fatty acids in adipocytes, due to the activation of the pro-oxidant enzyme nicotinamide adenine dinucleotide phosphate reduced form (NADPH) oxidase [218], which is involved in the formation of ROS [219]. Similarly, it has been observed that adiposity is associated with high plasma levels of lipoperoxidation due to an increase in 8-epi-prostaglandin F2α (8-epi-PGF2α), which leads to the appearance of complications, such as insulin resistance [220]. At the transcriptional level, it also has a negative impact, since it influences the gene expression of the shelterin complex, thereby altering the function of telomeres and triggering the inflammatory process and predisposition to chronic diseases [221].

On the other hand, when there are altered basal blood glucose levels or glucose intolerance, triglycerides (TG) levels and the 8-OHdG adduct are increased [222]. Likewise, an increase in TG, total cholesterol, LDL cholesterol (LDL-C), and MDA are associated with the decrease in the activities of the enzymes, GPx, and SOD in plasma [223].

Regarding the lipid profile, it is known that postprandial hypertriglyceridemia can generate OxS, predisposing to the appearance of chronic cardiometabolic diseases [224], while in animal models, it generates prediabetic neuropathy [225]. Furthermore, it has been shown that low levels of HDL-C are related to an increase in lipoperoxidation and hyperlipidemia in plasma [223,226]. It should be noted that this lipoprotein has been conferred anti-inflammatory and antioxidant effects [227].

OxS can modulate blood pressure levels, since ROS directly influence the vascular system through processes such as contraction and dilation or causing hypertension through signal transduction pathways mediated by changes in the cellular redox state [228]. The formation of O_2_^●−^ has been pointed out as a primary factor for the development and evolution of hypertension, including insulin resistance [229]. Angiotensin II is a hormone that is also involved in increasing blood pressure, alongside an increase in PGF2α that exerts antinatriuretic and vasoconstrictor effects with endothelin production, which is involved in the vascular damage caused by OxS [230,231].

Therefore, it can be assumed that OxS is involved in each of the MetS components (Table 2), which in turn are associated with alterations in telomere length (Table 3) predisposing to the deterioration of the metabolic condition of the patients with MetS [134].

## 9. Telomere Length and Inflammation in the MetS

Telomere shortening, as mentioned above, is associated with many conditions including MetS, which also presents a low-grade inflammatory state [255]. In other words, it does not cause an injury or loss of the infiltrated tissue [256] that is associated with alterations in the circulating levels of cytokines and acute phase reactants. A vicious cycle appears to be created between the inflammatory process that contributes to telomere dysfunction and aging, while attrition can promote low-grade inflammation [257] and ultimately reflect some type of disability or decrease in life expectancy.

It has been proposed that an increase in body mass index (BMI) stimulates the release of inflammatory mediators such as tumor necrosis factor-alpha (TNF-α), interleukin 6 (IL-6), C-reactive protein (CRP), and fibrinogen. Thus, it is considered that a cumulative inflammatory load due to the combination of high levels of IL-6, TNF-α, and CRP is associated with greater probabilities of a short TL in older adults with MetS [241,246,258,259,260,261,262].

A transcription factor that plays a key role in the inflammatory process as well as in the regulation of the catalytic subunit of the telomerase is the nuclear factor-kB (NF-kB) [263]. Given the fact that, in an injury response, NF-kB is activated by pro-inflammatory signaling mediated by TNF-α, a robust endogenous ROS generator, via c-Myc inducing rapid translocation of NF-kB, which ultimately leads to the increased expression and activation of the TERT subunit of telomerase [264]. It has also been observed that when telomerase is overexpressed, the non-phosphorylated accumulation of NF-kB-p65 is induced in the nucleus, without affecting its signaling and probably increasing its stability by avoiding the recognition of degradation mechanisms mediated by the ubiquitin/proteasome system. In addition, its accumulation is positively associated with IL-6 gene expression [265,266]. It is also known that the expression of RAP1, a component of the shelterin complex, acts as a modulator of the NF-kB [267]. There is evidence that supports the fact that an increase in ROS levels is associated with an increase in the expression of the TNF-α and NF-kB. In addition, the activation of p53 leads to the induction of NF-kB dependent pro-inflammatory cytokines, which may precipitate the development of T2DM [268].

When an alteration in the homeostasis of pro-inflammatory and anti-inflammatory adipokines occurs, caused by obesity, it can induce insulin resistance and lead to the development of MetS [269]. Some molecules are altered in this syndrome, as they are associated with increased adiposity, as in the case of monocyte chemoattractant protein (MCP-1). On the contrary, other adipokines with anti-inflammatory properties such as adiponectin (ApN) are decreased [270]. The latter is related to inhibition of the expression of TNF-α in adipose tissue and the cells of the immune system such as macrophages [271]. A positive association has even been observed between TL and adiponectin, bringing positive effects against accelerated aging [272].

In aged animals, TERT gene expression is negatively correlated with weight, while TNF-α and MCP-1 have a positive correlation, suggesting that age and weight are factors that impact telomerase expression. This can generate an alteration in the function of telomeres in adipose tissue, thereby predisposing to an inflammatory state and age-related chronic diseases [221].

On the other hand, it is recognised that suppressing the chronic inflammatory process may be a key factor for greater longevity [273] and health. In this regard, the consumption of chayote reduces the concentration of TNF-α and maintains telomerase levels [189,274]. Meanwhile, telomerase activator (TA-65), a natural compound used in traditional Chinese medicine, can lengthen telomeres and reduce levels of inflammation mediated by TNF-α and CRP in patients with MetS [275,276].

## 10. Effect of Healthy Lifestyles on Oxidative Stress and the Telomere Length

The dynamics of telomere length can depend on the type of lifestyle that the person develops. For example, healthy habits include aerobic exercise such as running [277], which causes an increase in the expression of the catalytic subunit of the telomerase hTERT and the protein TPP1 [278]; Tai Chi practice also improves telomerase activity [279], which is probably attributable to an increase in the activity of endogenous SOD and GPx enzymes and a reduction of lipoperoxidation during aging [280,281]; and intensive activities such as skiing, badminton, or basketball are related to a much longer TL [282]. It should be noted that the exercise must be personalised, since the effects are variable according to the frequency, duration, type, intensity, and chronological age of the individual [283].

Similarly, nutritional status is another factor that influences telomere length [284]. For example, the long-term supplementation of foods that are rich in omega-3 (*n-3*) polyunsaturated fatty acids (PUFA) [285], vitamins such as vitamin A [286], B or folate [287], C, E [288], and D [289], carotenoids such as lutein and zeaxanthin [290,291], polyphenols [292], fiber [293], greater adherence to the Mediterranean diet, or a diet rich in vegetables and fruits (whole grains, nuts, tea, coffee, and legumes) or consumption of eggs, fish, seaweed, minerals such as copper, iron, magnesium, and calcium [294,295,296,297], delay the shortening of the TL in older adults through glycated hemoglobin (HbA1c) [284,288]. These effects are mainly attributable to the overexpression of endogenous antioxidants that cause a decrease in OxS and mitochondrial dysfunction [298] or they are also attributable to its anti-inflammatory properties [299].

Likewise, some supplements have been used for therapeutic purposes, such as resveratrol, a polyphenolic compound found in grapes, which increases telomerase activity by increasing the expression of the catalytic subunit hTERT in a dose-dependent manner in patients who suffered from myocardial infarction [300] and improved glucose (glycated hemoglobin) and insulin levels in middle-aged subjects with T2DM [301], conferring hypoglycaemic, antioxidant, and anti-inflammatory properties [302]. Similar properties have been attributed to chayote, which maintains telomerase levels associated with an increase in SOD and a decrease in OxS together with a decrease in TNF-α in patients with MetS [189,274]. Additionally, in vitro studies have shown that turmeric roots cause an increase in TERT activity, which can have a favorable effect on telomere length [303].

Unhealthy lifestyles include sleep deprivation and its disorders, such as insomnia [304] and obstructive sleep apnea [305], which have considerable adverse effects during aging that are associated with increased lipoperoxidation [306] and with telomeric attrition compared to older adults with longer periods of sleep (>7 h per night) [307,308]. A similar effect is observed in psychological stress in terms of OxS and TL, also showing lower telomerase activity [309]. Regarding smoking, there are contradictory results over the TL [310]; however, most studies suggest that there is a shortening of telomeres in smokers [311] associated with lipoperoxidation [312]. In addition, the consumption of sugary carbonated beverages is associated with shorter telomeres [313], even if the consumption starts from the early stages of life and before the development of obesity [314]. Similarly, a higher intake of processed meat or heavy alcohol consumption may result in the reduction of the TL [315,316]. Hence, malnutrition with a high intake of fats, sugars, and sodium, along with a low intake of fruits and vegetables, predisposes to constant OxS levels, thereby accelerating the appearance of MetS.

## 11. Conclusions

MetS is characterised by a series of different metabolic abnormalities associated with OxS and decreased antioxidant protection mechanisms. This predisposes to telomere length shortening, which in turn is aggravated by the cumulative effects of its components, such as obesity, hyperglycemia, and hypertension, while dyslipidemia shows a discrepancy in these associations. The difficulty for the correct treatment lies in its multifactorial nature and in the use of drugs that are used simultaneously for long periods and with possible side effects. Therefore, new interventions are necessary, such as promoting healthy lifestyles with physical activity, the use of nutraceuticals, senolytics or inhibitory antisense oligonucleotides. Such interventions delay the telomeric attrition and decrease or suppress the oxidative state, thus helping to respond on time to the control of the different conditions of the MetS to prevent the development of chronic degenerative diseases and improve the living conditions of these people.

## Figures and Tables

**Table 1:** MetS and its association with telomere length.

Population with MetS	Determinations	Objective	Findings	Ref.
7370 patients (56–73 years old).	Evaluation of the components of the MetS. The average TL in leukocytes was determined by qPCR.	To investigate gender differences and their association between TL and MetS.	An increase in the number of MetS components is associated with shorter TL in the female population.	[211]
2842 patients (18–65 years old). Follow-up for 6 years	Evaluation of the components of the MetS. Basal TL in leukocytes was determined by qPCR.	To associate TL with the metabolic profile and with the MetS components.	Short TL is associated with higher MetS component scores, which persist even after 6 years.	[212]
34 male patients (55–68 years old).	Evaluation of the components of the MetS. The TL in leukocytes was determined by TRF. The bilateral ELC was determined by observation of a deep cut in both ears through the earlobe.	To determine if the ELC is related to telomeric shortening.	Bilateral ELC is a dermatological indicator associated with excessive telomere loss in patients with MetS.	[213]
400 women (18–86 years old).	Evaluation of the components of the MetS. The TL in leukocytes was determined by qPCR.	To determine the TL and its association with the metabolic condition in obese women.	TL is related to MetS and with a greater number of metabolic abnormalities.	[214]
115 subjects (43–87 years old)	The TL in leukocytes was determined by qPCR. Test 2hPG.	To establish the relationship between TL with the different components of MetS, glucose tolerance, and age.	MetS is associated with shorter telomeres. For its part, the 2hPG level showed a relationship with TL regardless of the presence of MetS.	[215]
1808 patients (18–65 years old) Follow-up for 6 years	The TL in leukocytes was determined by qPCR. Anthropometric and biochemical parameters.	To determine whether the components of the MetS predict TL through time and if the alterations are parallel to telomeric attrition.	An increase in waist circumference and glucose, as well as low HDL-C concentrations, are associated with shorter TL.	[216]
8074 patients (28–75 years old) Follow-up for 10 years	The TL in leukocytes was determined by qPCR. Anthropometric and biochemical parameters.	Evaluate the dynamics of TL and identify the factors associated with temporal changes in TL.	Conditions associated with MetS are factors that accelerate telomere attrition.	[217]

MetS: metabolic syndrome; TL: telomere length; qPCR: quantitative PCR; TRF: telomere restriction fragment analysis; ELC: diagonal earlobe crease; 2hPG: glucose tolerance reflected in 2-h post-load plasma glucose levels; HDL-C: high-density lipoprotein-cholesterol.

**Table 2:** Components of MetS and its association with telomeric length and OxS.

Components of MetS	Population	Determinations	Objective	Findings	Ref.
Obesity and OxS	59 subjects: (26–57 years old) CTR (n = 20); Obese (n = 22); Non-obese T2DM (n = 10) and Obese-T2DM (n = 7).	TL was determined by qPCR. Subcutaneous and visceral adipose tissue from subjects undergoing abdominal surgery. The size of the adipocytes was determined by histological staining. Lipid peroxidation by fluorometry.	To determine the association between adipocyte size and adipose tissue TL.	There is hypertrophy in adipocytes of obese, T2DM, and obese-T2DM subjects related to shortened TL. TBARS levels were higher in obese-T2DM and T2DM.	[232]
Hypertension, insulin resistance, and OxS	327 men: (40–89 years old).	The TL in leukocytes was determined by TRF. Determination of HOMA-IR.	To determine the association of TL with insulin resistance, OxS, and hypertension.	Hypertension, increased insulin resistance, OxS, and age are associated with shorter TL, being more evident in hypertensive patients, largely due to insulin resistance.	[233]
Hyperglycemia and OxS	120 subjects: (38–71 years old) CTR, with IGT, T2DM y T2DM^−^ atherosclerosis.	The TL in leukocytes was determined by TRF. Levels of TBARS, PCO, and CRP were measured by standard methodologies. IMT was assessed by ultrasonography.	To evaluate if the TL shortening occurs in the IGT stage and if it is greater in subjects with T2DM and atherosclerosis.	TL is lower in patients with T2DM and atherosclerosis. IGT and TL were negatively correlated with TBARS, PCO, and IMT. T2DM and TBARS are significant determinants of shortening.	[234]
Hyperglycemia and OxS	21 subjects: (50–65 years old) with T2DM.	TL was determined in monocytes by FISH. Oxidative damage by flow cytometry.	To establish if telomere shortening characterises T2DM.	TL in the diabetic group was lower and was associated with elevated levels of 8-oxoguanine.	[235]
Hyperglycemia and OxS	80 subjects: (49–56 years old) T2DM (n = 40) CTR (n = 40)	Lymphocyte TL was determined by TRF. Determine MDA plasma levels using TBARS.	To determine whether telomeric shortening occurs in T2DM patients.	TBARS levels showed a negative correlation with shortened telomeres in subjects with T2DM.	[236]
Hyperglycemia and OxS	621 patients: T2DM (n = 173) (24–92 years old) CTR (n = 448) (18–61 years old).	The TL in leukocytes was determined by qPCR. TAOS was determined by a photometric microassay. The patients were also genotyped for the UCP2 functional variants −866G> A and A55V.	To determine the association between TL and T2DM, OxS, and gene variation in UCP2.	The shorter TL was associated with T2DM attributed to high OxS. Carriers of the UCP2 -866A allele have a shorter TL compared to common homozygotes.	[237]

MetS: metabolic syndrome; OxS: Oxidative Stress; CTR: control; TL: telomere length; qPCR: quantitative PCR; T2DM: Type 2 diabetes mellitus; TBARS: levels of thiobarbituric acid reactive substances; TRF: telomere restriction fragment analysis; HOMA-IR: homeostatic model assessment insulin resistance; IGT: impaired fasting glucose; PCO: protein carbonyl content; CRP: C-reactive protein; IMT: carotid intima-media thickness; FISH: fluorescent in situ-hibridization); MDA: malondialdehyde; TAOS: total antioxidant status; UCP2: a gene involved in the mitochondrial production of ROS.

**Table 3:** Components of MetS and its association with telomeric length.

Components of MetS	Population	Determinations	Objective	Findings	Ref.
Obesity	309 participants (8–80 years old).	The average TL in leukocytes was determined by qPCR. Body fat was determined by DXA. The volume of adipose tissue was determined by MRI. Anthropometric indicators.	To evaluate the relationship between TL and adiposity.	Greater total and abdominal adiposity is associated with shorter TL, suggesting that obesity may accelerate the aging process.	[238]
Obesity	2721 subjects: (70–79 years old). Follow-up for 7 years.	TL in leukocytes was determined by qPCR. Adipose levels: BMI, % of body fat (DXA), and ACT scan to determine visceral and subcutaneous fat.	To determine if TL can be a risk factor for increased accumulation of adipose tissue.	The shorter TL can be a risk factor for adiposity.	[239]
Obesity	2912 women: (40–70 years old).	TL in leukocytes was determined by qPCR. Anthropometric indicators.	To determine the association between TL and anthropometric indices.	Telomere shortening is associated with obesity, the circumference of the waist and hips. The normal weight maintains the TL.	[240]
Obesity	3256 subjects: (14–93 years old).	The TL in leukocytes was determined by qPCR. Obesity indexes (BMI, waist circumference, % body fat, waist-hip ratio, and waist-height). High sensitivity CRP test.	To determine the association between TL and obesity rates.	TL is inversely associated with all obesity parameters and with CRP.	[241]
Obesity and insulin resistance	49 subjects: (21–43 years old). Follow-up for 10 years.	The TL in leukocytes was determined by TRF. Determination of HOMA-IR.	To determine if insulin resistance accelerates telomere attrition.	An increase in body weight and HOMA-IR is associated with a decrease in TL and with aging.	[242]
Obesity, TG, and hypertension	72 subjects: (45–60 years old).	The TL in leukocytes was determined by TRF. Subcutaneous adipose tissue samples were obtained from patients undergoing surgical procedures. Anthropometric and biochemical parameters.	To establish the relationship between TL in adipose tissue cells, with age and obesity.	TL was negatively associated with BMI, TG, and SBP in obese patients, which could contribute to their comorbidities.	[243]
TG and hyperglycemia	218 patients: (45–60 years old) T2DM (n = 142); CTR (n = 76).	TL was measured by qPCR. Biochemical and anthropometric data were collected.	To assess whether metabolic status contributes to premature aging.	TL was reduced in men with T2DM and inversely correlated with TG and total cholesterol.	[244]
TG	142 patients: (40–79 years old). Follow-up for 10 years.	TL was measured by qPCR. Biochemical and anthropometric data were collected.	To investigate the effects of bariatric surgery-induced weight loss on TL.	TL was inversely associated with baseline plasma TG and cholesterol concentrations.	[245]
TG, HDL-C, glucose, and blood pressure	7252 subjects: (20–84 years old)	The TL in leukocytes was determined by qPCR. Anthropometric and biochemical measurements.	To examine the associations between TL and 17 cardiovascular biomarkers.	TL was inversely associated with BMI, waist circumference, % fat, TG, blood pressure, and CRP and positively with HDL-C	[246]
TG and HDL-C	360 patients: (18–70 years old)	The TL in leukocytes was determined by qPCR. Anthropometric and biochemical measurements.	To determine the association between TL and coronary risk factors.	There is no association between TL and coronary risk factors, including cholesterol, TG, and HDL-C	[247]
HDL-C	6468 patients: (19–85 years old)	The TL in leukocytes was determined by qPCR. Anthropometric and biochemical measurements.	To investigate whether lipoproteins are associated with TL.	TL is not associated with LDL-C and TG but is positively associated with HDL-C when telomere length is shorter.	[248]
HDL-C	8892 subjects: (41–42 years old)	TL was measured by qPCR. Anthropometric and biochemical measurements were made.	To determine the relationship of TL with cardiometabolic risk profile.	A positive association was found between HDL-C and TL.	[249]
Hypertension	163 men: (60–64 years old).	Lymphocyte TL was determined by TRF. Extracranial carotid plaques were evaluated by ultrasonography.	To examine the relationship between TL and atherosclerotic plaques with the presence of hypertension	A shorter TL is associated with a greater predisposition to carotid artery atherosclerosis	[250]
Hypertension	3097 subjects: (23–76 years old) Hypertensive (n = 1415)	Meta-analysis The TL in leukocytes was determined by qPCR and TRF.	To determine if TL is related to hypertension	Telomeres are shorter in hypertensive than in normotensive individuals.	[251]
Hypertension	767 subjects: (30–80 years old). CTR (n = 379) Hypertensive (n = 388)	The relative length of the telomeres of the leukocytes was determined by qPCR	To investigate the association between TL and the risk and prognosis of hypertension	TL is significantly lower in patients with hypertension than in normotensive subjects.	[252]
Hypertension	98 twins: (18–44 years old)	The TL in leukocytes was determined by TRF. Anthropometric measurements	To investigate the relationship between TL and pulse pressure.	TL showed an inverse relationship with pulse pressure.	[253]
Hyperglycemia	272 subjects: (61–76 years old) CTR (n = 104) T2DM (n = 103) T2DM + MI (n = 65)	The TL in leukocytes was determined by qPCR. Glycaemic control markers: HbA1c, glucose, and waist–hip ratio.	To determine TL and its association with glycaemic control.	Patients with T2DM + MI have shorter TL than subjects with T2DM and CTR. Glycaemic control markers showed an inverse correlation with TL.	[254]

MetS: metabolic syndrome; TL: telomere length; qPCR: quantitative PCR; DXA: dual-energy X-ray absorptiometry; MRI: magnetic resonance imaging; BMI: body mass index; CTR: control group; CRP: C-reactive protein; TRF: telomere restriction fragment analysis; HOMA-IR: homeostatic model assessment insulin resistance; TG: triglycerides; SBP: systolic blood pressure; T2DM: type 2 diabetes mellitus; HDL-C: high-density lipoprotein-cholesterol; LDL-C: low-density lipoprotein-cholesterol; MI: myocardial infarction; HbA1c: glycated hemoglobin.

## Data Availability

Not applicable.
